# Genome-wide identification and expression of *SAUR* gene family in peanut (*Arachis hypogaea* L.) and functional identification of *AhSAUR3* in drought tolerance

**DOI:** 10.1186/s12870-022-03564-2

**Published:** 2022-04-07

**Authors:** Yiyang Liu, Lina Xiao, Jingxian Chi, Rongchong Li, Yan Han, Feng Cui, Zhenying Peng, Shubo Wan, Guowei Li

**Affiliations:** 1 Key Laboratory of Crop Genetic Improvement & Ecology and Physiology, Institute of Crop Germplasm Resources, Shandong Academy of Agricultural Sciences, Ji’nan, 250100 Shandong Province China; 2 Key Lab of Plant Stress Research, College of Life Sciences, Shandong Normal University, Ji’nan, 250014 Shandong Province China

**Keywords:** *SAUR* genes, drought stress, IAA, expression pattern, peanut

## Abstract

**Background:**

Small auxin-upregulated RNAs (*SAURs*) gene family plays important roles in plant growth, development, and stress responses. However, the function of few *SAUR* genes is known in the peanut (*Arachis hypogaea* L.), one of the world’s major food legume crops. This study aimed to perform a comprehensive identification of the *SAUR* gene family from the peanut genome.

**Results:**

The genome-wide analysis revealed that a total of 162 *SAUR* genes were identified in the peanut genome. The phylogenetic analysis indicated that the SAUR proteins were classified into eight subfamilies. The *SAUR* gene family experienced a remarkable expansion after tetraploidization, which contributed to the tandem duplication events first occurring in subgenome A and then segmental duplication events occurring between A and B subgenomes. The expression profiles based on transcriptomic data showed that *SAUR* genes were dominantly expressed in the leaves, pistils, perianth, and peg tips, and were widely involved in tolerance against abiotic stresses. A total of 18 *AhSAUR* genes selected from different subfamilies randomly presented 4 major expression patterns according to their expression characteristics in response to indole-3-acetic acid. The members from the same subfamily showed a similar expression pattern. Furthermore, the functional analysis revealed that *AhSAUR3* played a negative role in response to drought tolerance.

**Conclusions:**

This study provided insights into the evolution and function of the *SAUR* gene family and may serve as a resource for further functional research on *AhSAUR* genes.

**Supplementary Information:**

The online version contains supplementary material available at 10.1186/s12870-022-03564-2.

## Background

As a general coordinator of plant growth and development, the phytohormone auxin plays an essential role in controlling cell division, expansion, and patterning [1]. Dynamic changes in auxin levels require early response genes to trigger gene reprogramming precisely and rapidly, such as the auxin/indole-3-acetic acid (*Aux/IAA*) family, auxin response factor (*ARF*) family, small auxin-upregulated RNA (*SAUR*), aminocyclopropane-1-carboxylic acid synthase, glutathione-S-transferase, and auxin-responsive Gretchen H3 family [2–4]. Among these genes, the *SAUR* gene family is the largest family of early auxin response genes in higher plants, which have been implicated in regulating multiple biological processes [5]. On examining the functions of *SAUR* genes reported until recently, various *SAURs* were found to be involved in auxin synthesis and transport [6, 7], altering apical hook development [8, 9] and regulating hypocotyl and stamen filament elongation [10], leaf growth and senescence [11, 12], or root growth and development [13, 14]. Besides auxin, ethylene, brassinosteroid, gibberellin, abscisic acid (ABA), jasmonic acid, light, and osmotic stresses regulate the expression of *SAUR* genes [5, 15, 16], indicating that *SAURs* contribute to other hormonal- and environmental factor–mediated plant growth and development.

Cultivated peanut (*Arachis hypogaea* L.) is an important crop for human oil and food supply. The peanut is an allotetraploid species (2*n* = 4*x* = 40) arising from a hybridization between wild diploid species *A. duranensis* (AA) and *A. ipaensis* (BB) [17]. After fertilization, the gynophore of peanut forms a unique organ (known as the peg), which grows groundward and penetrates the soil [18]. After penetrating the soil, the tip of the peg gradually expands to develop a pod [19]. Previous studies have shown that auxin plays a major role in the growth of peanut stem, branch, flower, and peg, as well as leaf photosynthesis and metabolite transport in the pegging stage, which could be the physiological basis for peanut yield formation [20, 21].

Since then, *SAURs* have been found in many species, including *Arabidopsis* (79 members), rice (55 members), sorghum (71 members), maize (79 members), and other species [22–27]. However, the function of few *SAUR* genes is known in the peanut. The recently published peanut genome makes it more convenient for us to carry out the systematic analysis of *SAUR* [28, 29]. In this study, comprehensive genome-wide analyses of *AhSAUR* genes for systematic phylogenetic and evolutionary analyses, and expression characteristics in different tissues and under various stress treatments were examined. This study might provide a foundation for further studies on the potential functions of *SAURs* in the peanut.

## Results

### Identification, Classification, and Phylogenetic Analysis of the SAUR Genes in Peanut

**Fig. 1:**
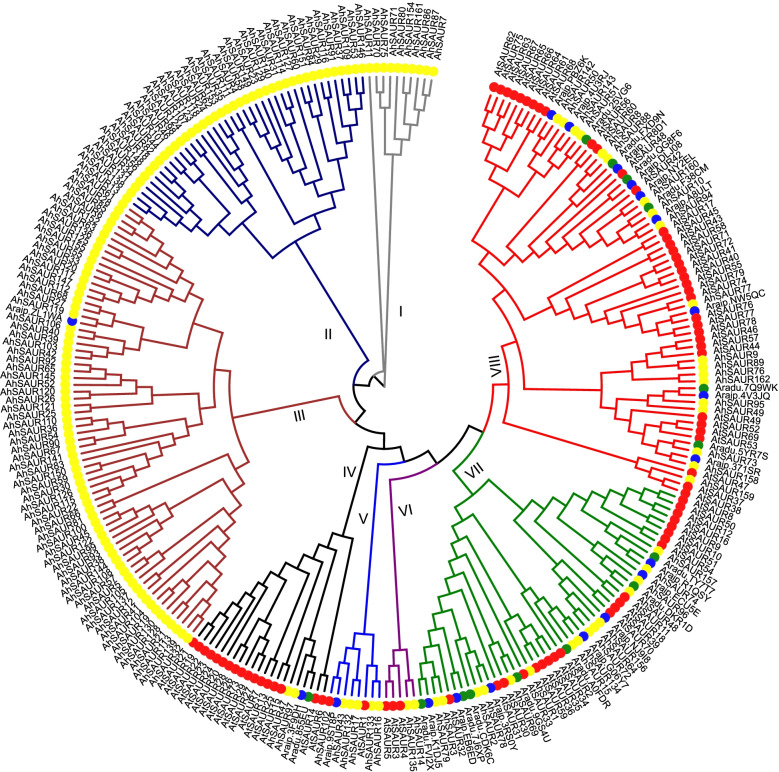
The phylogenetic relationship of SAUR family in Arabidopsis, *A. duranensis*, *A. ipaensis* and peanut. An unrooted maximum likelihood tree of SAUR family was constructed with 72, 14, 19 and 162 members in Arabidopsis, *A. duranensis*, *A. ipaensis*, and peanut, respectively. The SAUR members of Arabidopsis, *A. duranensis*, *A. ipaensis*, and peanut were marked by red, green, blue, and yellow circles, respectively. Bootstrap values from 1000 replicates are displayed at each node. Different subfamilies were marked with different branch colors

The genome of cv. Tifrunner available in PeanutBase (http://peanutbase.org/) was scanned to identify the *SAUR* family genes in the peanut using BLASTP and TBLASTN. The amino acid sequences of the known *SAUR* members in *Arabidopsis*, rice, and maize were used as queries. The BLAST results were then examined using the hidden Markov model with the SAUR domain. A total of 162 *SAUR* genes in the peanut were finally identified and designated as *AhSAUR1*–*162*, ordered by their location in chromosome from the top to bottom and from subgenome A to subgenome B (Fig. 1, Table S1). *SAUR* genes in the diploid ancestors of the cultivated peanut were also identified: *A. duranensis* and *A. ipaensis*. Surprisingly, only 14 and 19 members of the *SAUR* gene family were found in A and B diploid genomes, respectively (Table S2). This implied that the *SAUR* gene family was expanded and duplicated in the peanut after tetraploidization. All SAUR proteins from *Arabidopsis*, peanut, *A. duranensis,* and *A. ipaensis* were used to construct a phylogenetic tree so as to characterize the phylogenetic relationship among the *AhSAURs*. The SAUR proteins were classified into eight subfamilies (I–VIII). Subfamilies VII and VIII included the SAUR members from all four species, while the other subfamilies only contained members from one to three species. Especially in subfamilies I–III, only one member was from *A. ipaensis* and the rest were from the cultivated peanut.

### Chromosomal Location and Distribution of *AhSAURs*

**Fig. 2:**
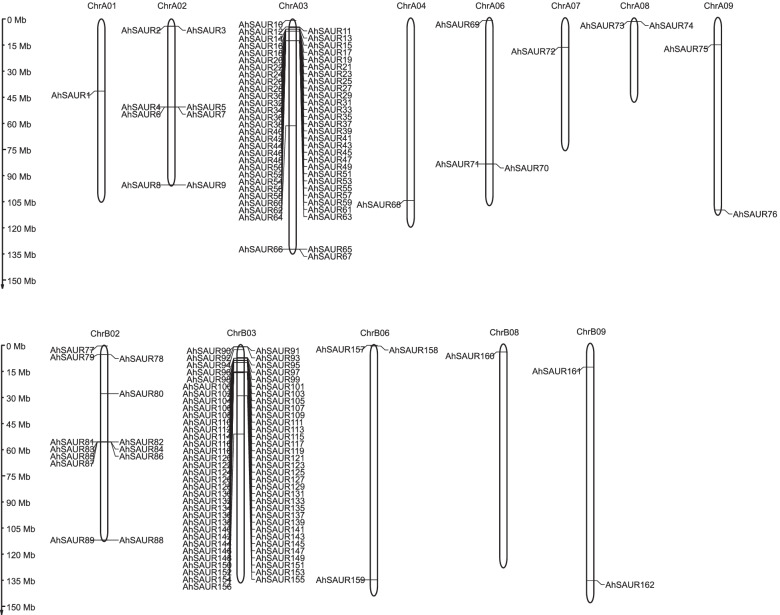
The chromosomal location and distribution of 162 *AhSAUR* genes. Chromosome size is indicated by its relative length. The scale bar represents megabases (Mb). The physical locations of *AhSAURs* are drawn on each chromosome. The figure was generated by the MapGene2Chrom

The chromosomal distribution of *AhSAURs* was further investigated according to their physical locations in the peanut genome (Fig. 2). Of these genes, 76 were located in subgenome A and 86 in subgenome B. Moreover, the distribution of *SAUR* genes in chromosome between A and B subgenomes was not symmetrical. For example, only one *AhSAUR* was located on each of the chromosomes 01, 04, and 07 of the A subgenome, but not on the corresponding chromosomes of the B subgenome. However, most *AhSAUR* genes were tightly packed into clusters and tandemly distributed on chromosomes (A02, A03 and B02, B03), presenting a high-density distribution on some chromosomal regions. This result was in line with a previous study that analyzed repeated events in *Arabidopsis* and other species, indicating that some *SAUR* subfamily members were most likely derived from repetitive events.

### Gene Duplication Event and Syntenic Analysis of *AhSAURs*

**Fig. 3:**
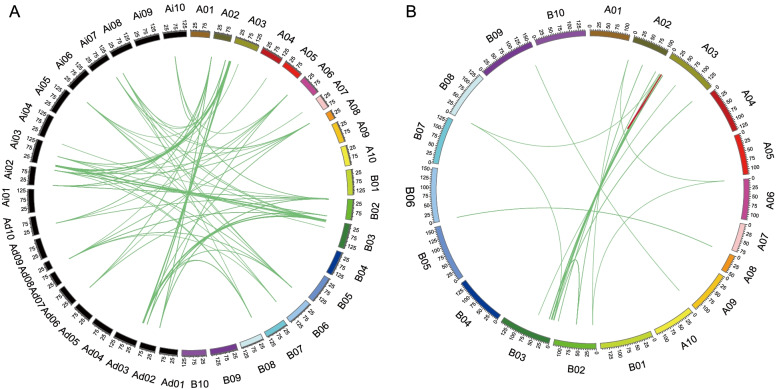
Gene duplication events and syntenic analysis of *SAURs* in three *Arachis* species. a: Synteny analysis of *SAUR* genes in *A. duranensis*, *A. ipaensis*, and peanut. The green lines indicate that these two syntenic genes from *A. duranensis* and peanut or *A. ipaensis* and peanut. The black box represents chromosomes of *A. duranensis* and *A. ipaensis.* The color box represents chromosomes of peanut*.* Green lines represent the syntenic relationships between *SAURs*. Ad, *A. duranensis*; Ai, *A. ipaensis*. b: Synteny analysis of *SAUR* genes in peanut. Green lines represent the syntenic relationships between *SAURs*. Red lines represent the gene pairs of tandem duplication. The scale bar represents megabases (Mb). The chromosome numbers are indicated on the top of each bar

Given that *AhSAURs* in the peanut are likely to have risen from a recent tandem duplication event and the number of *SAUR* genes in the peanut were significantly more than that in its diploid ancestors, synteny analysis was performed between the peanut and its diploid ancestors to gain further understanding of the origin and evolutionary relationship of *AhSAUR* genes (Fig. 3a). According to syntenic analysis, 73 and 93 syntenic gene pairs were detected between *A. duranensis* and the cultivated peanut, *A. ipaensis* and the cultivated peanut, respectively (Fig. 3b, Table S3). A single gene from the A or B diploid genome to form syntenic gene pairs with multiple genes from the cultivar peanut, indicating that the hybridization of A and B genomes and following tetraploidization led to the duplication of these genes. Furthermore, the gene duplication event inside the peanut genome was explored. A total of 113 syntenic gene pairs were identified in the peanut (Table S4). A number of tandem duplication events occurred in chromosome A03, but they were not found in B03. On the contrary, more segmental duplication events were observed in chromosome B03. Considering that a large number of *SAUR* genes were tandemly clustered on chromosome B03, it was hypothesized that the *SAUR* genes in A03 were first experienced in a recent tandem duplication event after the tetraploidization of the peanut, and then segmental duplication events occurred between A and B subgenomes. The results also implied that both tandem and segmental duplication events contributed to the expansion of *AhSAUR* genes in the tetraploid peanut.

### Transcriptome profiles of *AhSAURs* in Peanut

**Fig. 4:**
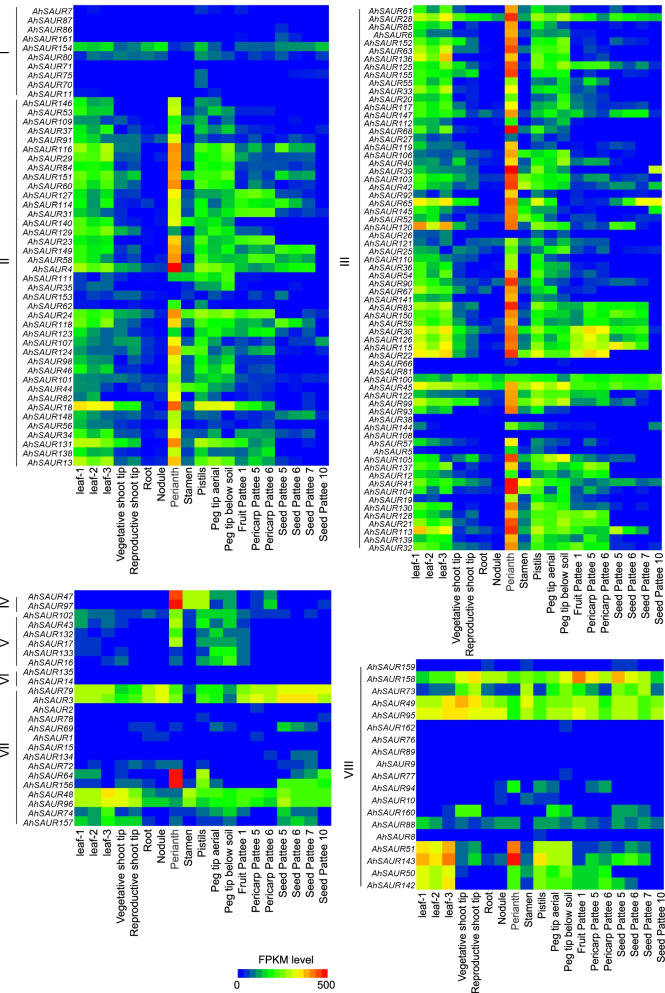
Heat map of expression profiles of all *AhSAUR* genes in 19 different tissues of peanut. The expression abundance of each transcript is represented by the normalized fragments per kilobase per million (FPKM) value and displayed as colored boxes from blue (lower expression) to red (higher expression)

The relative expression levels of the associated genes were analyzed from the RNA-seq datasets of 19 tissue samples that were previously investigated so as to study the expression patterns of *AhSAURs* in different tissues and developmental stages in the peanut (Table S5). In general, most of the *AhSAUR* genes had relatively higher expression levels in the leaves, pistils, perianth, and peg tips compared with the expression levels in other stages (Fig. 4). The gene expression levels of *AhSAURs* in subfamilies I and VI showed universally low expression in all developmental stages, except *AhSAUR154* that showed slightly higher expression in a few organs. *AhSAURs* belonging to subfamilies II and III showed dominant expression in the leaves, pistils, perianth, and peg tips. Most of the *AhSAURs* in subfamilies IV and V showed relatively higher expression in pistils, perianth, and peg tips that before and after the soil. However, the gene expression levels of *AhSAURs* in subfamilies I and VI showed the characteristics of two-level differentiation, that is, half of them had extremely low expression or even no expression in various tissues and the other half showed universally higher expression in all developmental stages.

Previous studies showed that some *SAUR* genes were involved in various abiotic stress responses in plants (Jain and Khurana, 2009; Kant et al., 2009). The expression patterns of *AhSAURs* were further studied under various stress conditions by analyzing the RNA-seq data for peanut seedlings. The results showed that the expression levels of most *AhSAUR* genes did not vary obviously under different stress conditions (Fig. S1). Only a few genes showed significant changes in the expression under different stress conditions. Of these genes, 4.3%/22.8% (7/37 out of 162), 4.3%/13.0% (7/21 out of 162), 14.8%/7.4% (24/12 out of 162), 24.7%/3.7% (40/6 out of 162), 4.3%/11.7% (7/19 out of 162), and 29.6%/11.1% (48/18 out of 162) were upregulated or downregulated under the ABA, H_2_O_2_, heat, NaCl, Polyethylene glycol (PEG), and low-temperature treatments, respectively. It was inferred that *AhSAURs* had a relatively stronger response to low temperature than other treatments. Moreover, most homologous gene pairs had similar expression levels, suggesting that they might perform similar physiological functions. For example, *AhSAUR3*/*79* were downregulated under ABA and PEG treatments and *AhSAUR*30/96 were significantly upregulated under low-temperature treatment.

### Expression Profiles of *AhSAURs* in Response to IAA

**Fig. 5:**
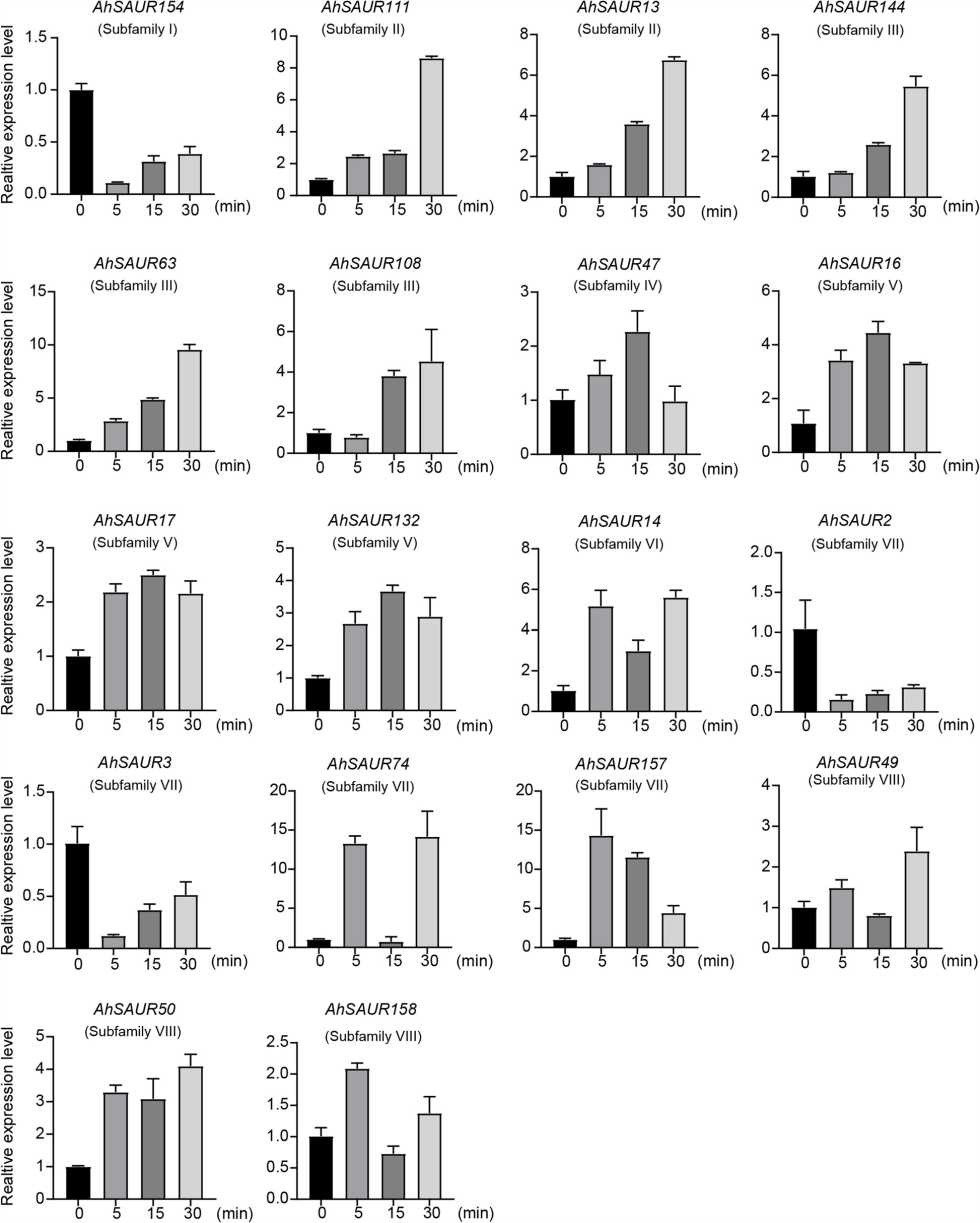
Expression analysis of *AhSAURs* under IAA treatments by qRT-PCR. The relative expression levels of 18 *AhSAURs* were tested by qRT-PCR. 0 min, 5 min, 15 min and 30 min indicate minutes after IAA treatment. The error bars show the standard deviation of three biological replicates

Previous studies showed that at least some *SAUR* genes could be induced by exogenous auxin. Some members (excluding which expression was not detected in leaves) from each subfamily were randomly selected to detect their expression characteristics under IAA treatment using quantitative reverse transcription–polymerase chain reaction (qRT-PCR) so as to further explore the potential function of *AhSAURs* in response to auxin (Fig. 5). In general, the members belonging to the same subfamily showed similar patterns of expression. Moreover, the expression patterns of these selected *AhSAURs* were divided into four major expression patterns according to their expression characteristics in response to IAA. *AhSAUR154* (subfamily I), *AhSAUR2* (subfamily VII), and *AhSAUR3* (subfamily VII) showed a dramatic downregulation with minimum levels in early 5 min, and then were upregulated gradually (in 15 and 30 min) under IAA treatment. The expression levels of the members of subfamily II (*AhSAUR111* and *AhSAUR13*) and subfamily III (*AhSAUR144*, *AhSAUR63*, and *AhSAUR108*) were gradually upregulated with the treatment time and reached a maximum in 30 min. The expression levels of the members of subfamily IV (*AhSAUR47*) and subfamily V (*AhSAUR16*, *AhSAUR17*, and *AhSAUR132*) showed an early (in 5 and 15 min) upregulation followed by a downregulation (in 30 min). The expression pattern of the remaining *AhSAUR14* (subfamily VI), *AhSAUR74* (subfamily VII), and members of subfamily VIII (*AhSAUR49*, *AhSAUR50*, and *AhSAUR158*) showed an early upregulation (at 5 min), then downregulation (at 15 min), and finally upregulation (at 30 min). In addition, *AhSAUR157*, with an expression pattern different from that of the other subfamily VII members, showed a dramatic rise to the highest level in 5 min, followed by a gradual decline in 15 and 30 min.

### Characterization of the Function of *AhSAUR3* in Response to Drought Stress

**Fig. 6:**
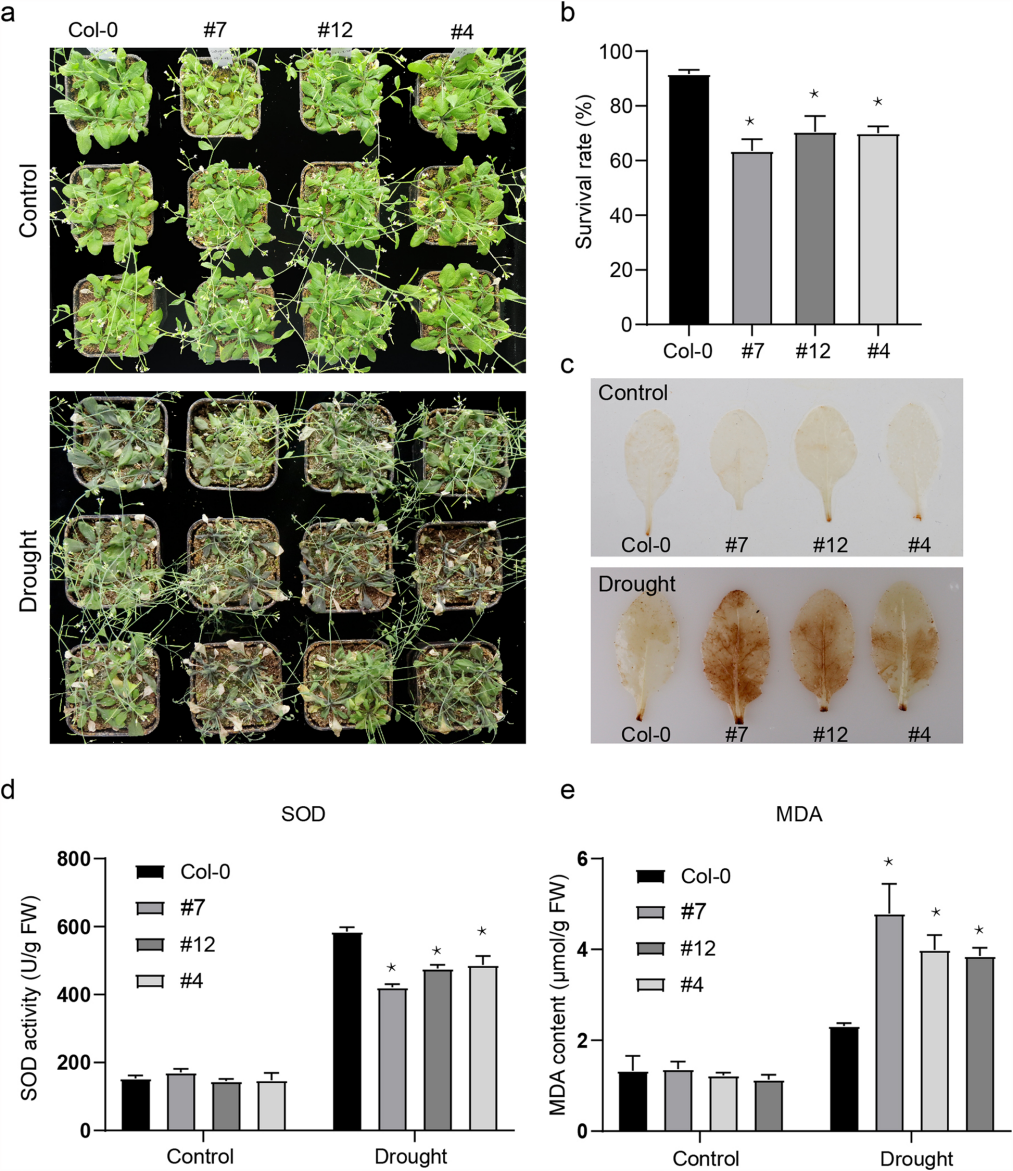
The *AhSAUR3*-overexpressed plants responded to drought stress. a: Physiological dehydration stress tolerance assay with *AhSAUR3*-overexpressed plants and wild-type (Col-0) subjected to severe drought stress without water for 14 d and then recovered for 4 d. b: Survival rates of wild-type (Col-0) transgenic and plants tested in a. c: 3, 3′-Diaminobenzidine (DAB) staining for H_2_O_2_ content in *AhSAUR3*-overexpressed plants and wild-type (Col-0). d: SOD enzyme activity in *AhSAUR3*-overexpressed plants and Col-0 under standard and salt stress conditions.e: MDA content in *AhSAUR3*-overexpressed plants and Col-0 under standard and salt stress conditions. All the data from at least three times was presented as mean ± standard error (SE). Asterisk indicates significant variation in transgenic lines compared with Col-0 by student’s *t*-test analysis with *p* < 0.05

*AhSAUR3* was selected for further analysis as it displayed high expression in all developmental stages and responded to ABA and drought treatment. Transgenic plants overexpressing *AhSAUR3* in *Arabidopsis* were generated under the 35S promoter to elucidate the biological function of *AhSAUR3*. Three independent *AhSAUR3*-OE (overexpression) transgenic lines (OE-4, OE-7, and OE-12; Fig. S2) were selected for further analysis. No obvious difference in morphology was found between (Col-0) and the three transgenic lines under normal conditions (Fig. 6a). The whole seedlings of 14-day-old wild-type (WT, Col-0) and transgenic plants were exposed to drought treatment for 2 weeks, followed by rehydration for 4 days, to evaluate the drought stress tolerance of *AhSAUR3*. Before drought treatment, the transgenic lines showed no significant difference in morphology compared with WT (Fig. 6a, upper panel). After drought treatment, the leaves of overexpressing lines exhibited an early withering phenotype, compared with other plants (Fig. 6a, lower panel). The *AhSAUR3*-overexpression lines OE-4, OE-7, and OE-12 showed lower survival rates (63.5, 70.5, and 70.0%, respectively) than WT (91.7%) (Fig. 6b). Dehydration promoted rapid accumulation of reactive oxygen species (ROS), such as H_2_O_2_, which functioned as a signal to trigger diverse acclimation responses to stress [30]. Therefore, whether *AhSAUR3* played a role in stress tolerance through detoxification of ROS was examined. The leaves of transgenic and WT lines were stained with 3,3′-diaminobenzidine (DAB) to detect H_2_O_2_ levels. As shown in Fig. 6c, under normal conditions, very few DAB-stained spots were observed in the leaves of all plant lines. After drought treatment, the leaves of *AhSAUR3-*overexpressing lines exhibited much stronger DAB staining than those of the corresponding control plants. In addition, the leaves from *AhSAUR3*-overexpressing seedlings showed higher superoxide dismutase enzyme activity and lower malondialdehyde contents compared with those from WT during dehydration (Fig. 6d and e). Taken together, these results demonstrated that the overexpression lines of *AhSAUR3* had a decreased resistance to drought stress in Arabidopsis.

## Discussion

In this study, 162 *SAUR* genes were identified in the cultivated peanut genome. Based on the phylogenetic analysis, these *AhSAURs* were divided into eight subfamilies. Notably, the members from subfamilies II and III could be due to gene expansion after tetraploid peanut formation (Fig. 1). Compared with their diploid A and B genomes with 14 and 19 genes, both tetraploid A and B subgenomes showed 5.8- and 4.5-times expansion, respectively, in their gene content (containing 76 and 86 genes, respectively). By gene duplication events and syntenic analysis, we found that both the possible chromosomal rearmaments and duplication events contributed to the expansion of *AhSAUR* genes in the tetraploid peanut after the hybridization of A and B genomes and following tetraploidization. The A subgenome played a dominant role in the *AhSAUR* gene expansion, implying active gene duplications after tetraploidization. Furthermore, it was proved based on the syntenic analysis of *AhSAURs* that the gene duplication events occurred in the A subgenome but not in the B subgenome (Fig. 3). Interestingly, *ARF* genes, such as *SAUR*, belonging to the early auxin response gene family, also experienced gene expansion events, with 114, 28, and 28 members in the peanut, *A. duranensis*, and *A. ipaensis*, respectively [28].

The expression patterns in 19 tissues of the cultivated peanut were investigated to further explore the potential functions of *AhSAURs* (Fig. 4). Most of *AhSAURs*, especially the new expanded members after tetraploidization (subfamily II), showed dominant expression in the leaves, pistils, perianth, and peg tip. Considering that the tetraploid peanut had larger leaves and flowers than its wild diploid peanut [28], it was inferred that *AhSAURs* played an important role in controlling the size of leaves and perianth. Increasing evidence from overexpression studies revealed that *SAUR* activity could induce growth in leaves, stems, and floral organs [23]. In recent years, the characterization of the *SAUR* family in many species*,* revealed that the different *SAUR* genes exhibited specific expression patterns throughout plant development. Meanwhile, the expression data in this study also demonstrated that some *AhSAUR* genes showed specific expression in the period of peg tip up or below the soil, which was considered to have played a key role in the restart of the peanut embryo. Previous studies demonstrated that the dark stimulus of the peg entering the soil was important for successful pod development, but light inhibited the formation of pods [18, 19]. Indeed, many recent studies revealed that the *SAUR* expression level was regulated in a light and circadian manner. For example, PIF3 conferred light signal responsiveness by repressing TCP4-induced activation of the *SAUR* genes in the dark, which induced their expression in dark-grown hypocotyls [18, 19]. Therefore, in this study, it was speculated that the *AhSAUR* genes specifically expressed in the peg before and after soil might be involved in the light regulation of pod development. In addition, several genes, such as *AhSAUR3*, *AhSAUR48*, and *AhSAUR95*, showed a constitutive expression pattern (Fig. 4), implying that these genes were more likely to play critical roles in regulating development during the whole growth period of the peanut.

*SAUR* genes are important gene families in auxin signaling transduction and are commonly used as early auxin-responsive markers. The levels of *AhSAUR* expression under IAA treatment were investigated using qRT-PCR. Overall, except subfamilies I and VII showing a dramatic downregulation in the early 5 min, the other subfamily members exhibited upregulation at this time (Fig. 5). This result implied that these members could be considered as auxin response genes. Moreover, the expression of different sets of SAUR genes could be positively or negatively regulated by many different stresses, including H_2_O_2_, heat, NaCl, PEG, and low temperature. Especially, *AhSAURs* had a relatively stronger response to low temperature than other treatments. These findings indicated that *AhSAUR* might not be involved only in auxin response but also widely involved in tolerance to abiotic stresses. Similar results have also been reported in other species [27].

According to the expression results, *AhSAUR3* displayed high expression in all developmental stages but was downregulated after ABA and PEG treatments. Therefore, it was speculated that the stress-induced downregulation of *AhSAUR3* probably compensated the plant’s investment in resistance mechanisms when the peanut suffered drought or oxidative stresses. The biological function of *AhSAUR3* was further investigated using transgenic Arabidopsis plants (Fig. 6). The results demonstrated that the overexpression lines of *AhSAUR3* had a decreased resistance to drought stress in *Arabidopsis*. This result also confirmed the previous conjecture. However, a recently published report showed that the overexpression of *AtSAUR32* (homologue of *AhSAUR3* in *Arabidopsis*) resulted in an increase in drought resistance of plants [31]. This opposite result implied that the *SAUR* genes in the peanut and *Arabidopsis* underwent a great divergence in their functions during the evolutionary process.

## Conclusions

Since the *SAUR* gene family was first verified to be involved in plant development, many studies identified the *SAUR* gene family in various plant species. In this study, 162 *SAUR* genes were identified in the peanut genome. The phylogenetic analysis indicated that the SAUR proteins were classified into eight subfamilies. The *SAUR* gene family experienced a remarkable expansion after tetraploidization, which contributed to the tandem duplication events first occurring in subgenome A and then segmental duplication events occurring between A and B subgenomes. The expression profiles showed that most *SAUR* genes had specific expression in the leaves, pistils, perianth, and peg tip, and were widely involved in tolerance of abiotic stresses. *AhSAUR* genes selected from different subfamilies randomly presented four major expression patterns in response to IAA. The members from the same subfamily showed similar expression patterns. Furthermore, the transgenic identification of *AhSAUR3* revealed that *AhSAUR3* played a negative role in response to drought tolerance. This study provided insights into the evolution and function of the *SAUR* gene family in the peanut and might help further understand the function of *AhSAUR* genes.

## Methods

### Identification of Peanut *SAURs* Genes

The SAUR genes of Arabidopsis (72 members), rice (55 members) and maize (79 members) were employed as queries to search against the reference genome of *Arachis hypogaea* cv. Tifrunner and its two ancestral diploid species, *A. duranensis*, and *A. ipaensis*, which available in the peanut genome database (http://peanutbase.org/) using BLASTP and TBLASTN analysis (score > 50, E-value<0.01) [32]. The SAUR family protein sequences of Arabidopsis were downloaded from Arabidopsis Information Resource website (TAIR, http://www.arabidopsis.org). The SAUR family protein sequences of rice and *Zea mays* were downloaded from the Phytozome 12 database (https://phytozome.jgi.doe.gov). Reciprocal BLASTP analysis was performed using NCBI to ensure that the subject hits most closely matched the SAUR family query. The BLAST results were finally examined using the hidden Markov model with the SAUR domain.

*Multiple Sequence Alignment, Phylogenetic Analysis and Chromosome Localization.*

Protein multiple sequence alignment was performed using software Clustal X 2.0 [33] and the phylogenetic trees were constructed using MEGA 5 with protein sequences, applying the maximum likelihood (ML) method with a bootstrap test of 1000 replications [34]. The chromosome location of *AhSAURs* were mapped with localization MapGene 2Chromosome V2 (http://mg2c.iask.in/mg2c_v2.0/).

### Detection of Orthologous Gene Pairs and Synteny Analysis

MCScanX v0.8 software (https://github.com/wyp1125/MCScanX) was used to detect the duplicated genes within peanut genome and the syntenic blocks among peanut, *A. duranensis*, and *A. ipaensis* [35]. Whole-genome protein sequences from peanut were merged and searched against themselves using BLASTP with an E-value cutoff of 1 × 10^−5^, then, the default parameters of MCScanX and associated downstream tools [32]. The relationships of the orthologous pairs among the two species were plotted using Circos (http://circos.ca/) [36].

### Plant Growth and Treatments

The peanut cultivar cv. Tiffrunner provided by the Shandong Institution of Peanut in Shandong, China (http://www.sdshss.com/), was used as the experimental materials in this study. After germination in sand for 8 days, peanut seedlings were transferred to hydroponic pots containing 2 L of Hoagland’s nutrient solution and grown in an artificial climate-controlled chamber with 16 h light (200 μmol protons m^−2^ s^−1^, 26 °C) and 8 h darkness (24 °C) at 50% relative humidity. The nutrient solution was changed weekly. 30 days old seedlings were used for IAA treatment. For IAA treatment, the seedling leaves were sprayed by 10 μM IAA solution, and then sampled at 0, 5, 10, 30, 60 min intervals [26]. The experiment was repeated three times. The samples were frozen in liquid nitrogen and stored at −80 °C for the following experiments.

### RNA-seq Data and Expression of SAUR Genes

To further characterize the function of peanut *SAUR* genes during peanut development, RNA-seq data from different tissues in cultivated peanut were downloaded from the National Center for Biotechnology Information (http://www.ncbi.nlm.nih.gov/) under BioProject PRJNA291488 [37]. A description of the peanut tissues is listed in Table S6. The reads were then aligned to the cultivated peanut reference genome (Tifrunner. gnm2. KVY3, https://www.peanutbase.org/data/public/Arachis_hypogaea/) by using HISAT2 software (v2.1.0) with default parameters [38]. Transcription sequence assembly and normalization of gene expression (fragments per kilobase million, FPKM) were performed using StringTie (v1.3.4, default parameters). RNA-seq data for various abiotic stresses in cultivated peanut were obtained in our previous study, which deposited in the National Center for Biotechnology Information (http://www.ncbi.nlm.nih.gov/) under BioProject PRJNA553073 [39]. The seedlings of FH1 used for sequencing were cultivated in 1/2 MS medium at room temperature for 10 days and were then treated with 2% NaCl for 24 h, 20% polyethylene glycol (PEG) 6000 for 24 h, low temperature (4 °C) for 24 h, heat (37 °C) for 24 h, 75 μM ABA for 24 h, 1% H_2_O_2_ for 24 h. RNA-seq data were analyzed by HISAT2 and Cufflinks to obtain FPKM (fragments per kilobase million) values [39]. The expression pattern of the *AhSAURs* genes in different tissues and under various abiotic stresses was generated with Heml 1.0 heatmap illustrator [40].

### RNA Extraction and qRT-PCR Analysis

All RNA samples were extracted using TIANGEN RNAprep pure Plant Kit (Tiangen, China) according to the manufacturers’ protocols and reverse transcribed with a PrimeScript RT Reagent Kit with gDNA Eraser (TaKaRa). qRT-PCR was performed in three independent biological replicates with SYBR Premix Dimer Eraser (TaKaRa). The data of relative gene expression was analyzed using the 2^-ΔΔCT^ method. Primers were designed by Primer-BLAST (https://www.ncbi.nlm.nih.gov/tools/primer-blast/). The primers sequences are listed in Table S7. *AhTUA5* gene expression was used as a constitutive control.

### Transformation and Drought Tolerance Assay

The full-length ORF *AhSAUR3* was amplified and constructed to PHB vector and transformed to Col-0 as previously described [41]. The RT-PCR was employed to investigate *AhSAUR3* expression in the transgenic lines (Table S7). Plants were grown under normal conditions (16 h of light/8 h of dark, 80 μE s^−1^ m^−2^ light intensity) at 22 °C. For drought tolerance assay, the whole seedlings of 14-day-old wild-type (Col-0) and transgenic plants were exposed to drought for 2 weeks, followed by rehydration for 4 days. Then, the survival rates were measured between WT and *AhSAUR3* transgenic lines.

### DAB Staining, SOD Enzymes Activities, and MDA Content

The method for DAB staining was according to previous study [42]. Firstly, the leaves of transgenic lines and wild-type plants were collected and then soaked in DAB solution (1 mg/mL DAB in 10 mM sodium phosphate buffer). Secondly, the leaves were treated with DAB solution for 24 h with gentle shaking in the darkness. Finally, the leaves were washed using absolute ethyl alcohol, transferred to 100 °C for 10 min.

The activity of SOD and the content of MDA was measured as previous methods [41]. Briefly, 0.3 g of leaves were sampled and kept in 1.8 mL phosphate-buffered saline (PBS; 0.1 M, pH 7.4) on ice. The crude extract was centrifuged at 10,000 g for 10 min at 4 °C. The MDA content was detected using Plant Malondialdehyde (MDA) assay kit (Colorimetric method) (Nanjing Jiancheng Bioengineering Institute, Jiangsu, China) at 532 nm in microplate reader. The SOD enzymes activity was determined by Superoxide Dismutase (SOD) assay kit (WST-1 method) (Nanjing Jiancheng Bioengineering Institute, Jiangsu, China). All the experiments were repeated at least three times and data were measured as the mean ± standard error (SE). And Student’s *t*-test with *p* < 0.05 was used for data variation.

## Supplementary Information

**Additional file 1.**

**Additional file 2.**

**Additional file 3.**

## Data Availability

All needed genome sequences and genome annotation files of *Arachis hypogaea* cv. Tifrunner and its two ancestral diploid species, *A. duranensis*, and *A. ipaensis* were obtained from PeanutBase (http://peanutbase.org/), and the published SAUR sequences of *A. thaliana* were acquired from the TAIR database (http://www.arabidopsis.org/). The transcriptome sequencing data of different tissues used in this study was got from previous report (https://www.ncbi.nlm.nih.gov/bioproject/?term=PRJNA291488). The transcriptome sequencing data of various abiotic stresses used in this study has been downloaded from previous report (https://www.ncbi.nlm.nih.gov/bioproject/?term=PRJNA553073). All databases in this study are available to the public.

## Abbreviations

SAUR: Small auxin-upregulated RNA
IAA: Indole-3-acetic acid
FPKM: Fragments per kilobase per million
ABA: Abscisic Acid
PEG: Polyethylene glycol
H_2_O_2_: Hydrogen peroxide
WT: Wild type
ROS: Reactive oxygen species
DAB: 3,3′-diaminobenzidine
SOD: Superoxide dismutase
MDA: Malondialdehyde
