# Supplementary material for: Genome-wide identification and expression of SAUR gene family in peanut (Arachis hypogaea L.) and functional identification of AhSAUR3 in drought tolerance
Source: BMC Plant Biol. 2022 Apr 7;22:178. doi: 10.1186/s12870-022-03564-2 (PMC8988358; doi:10.1186/s12870-022-03564-2)
Supplement: Supplementary file 1 — Additional file 1. [file 12870_2022_3564_MOESM1_ESM.zip › Figure S2. Identification of AhSAUR3 overexpressed lines in Arabidopsis..pdf]

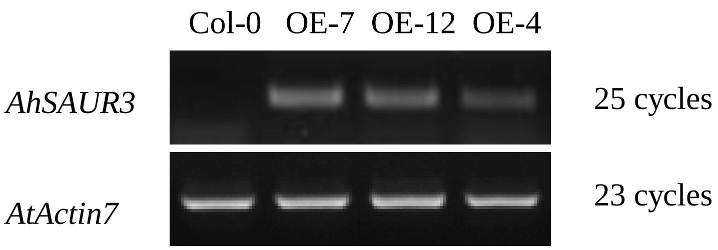

Figure S2. Identification of *AhSAUR3* overexpressed lines in Arabidopsis. Expression of *AhSAUR3* in the transgenic Arabidopsis, tested with RT-PCR. *AtActin7* was used as an internal control.
