# Supplementary figures and images for: Genome-wide identification and expression of SAUR gene family in peanut (Arachis hypogaea L.) and functional identification of AhSAUR3 in drought tolerance
Source: BMC Plant Biol. 2022 Apr 7;22:178. doi: 10.1186/s12870-022-03564-2 (PMC8988358; doi:10.1186/s12870-022-03564-2)

Original, uncropped DNA gel image of RT-PCR detection of *AhSAUR3* in Col-0, OE-7, OE-12, OE-4 in Figure S2.

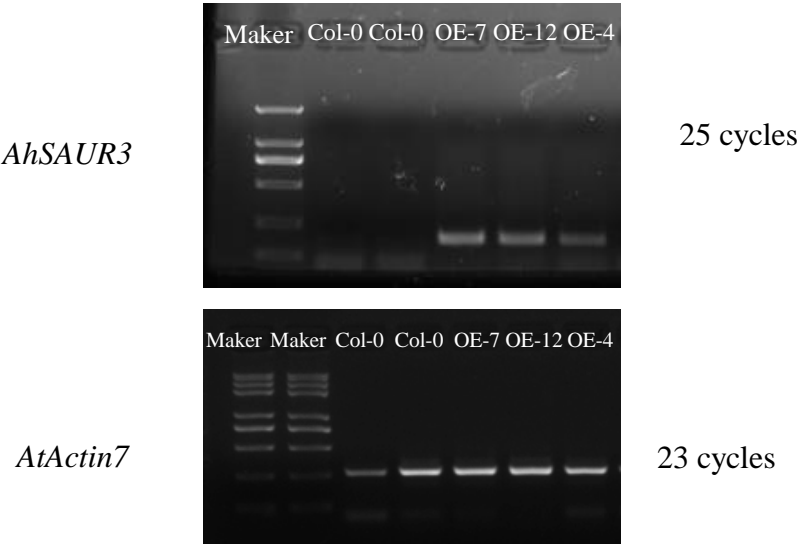

Supplement: Supplementary file 3 — Additional file 3. [file 12870_2022_3564_MOESM3_ESM.zip › Original, uncropped DNA gel image from Figure S2.pdf]
