# Supplementary material for: Genome-wide identification and expression of SAUR gene family in peanut (Arachis hypogaea L.) and functional identification of AhSAUR3 in drought tolerance
Source: BMC Plant Biol. 2022 Apr 7;22:178. doi: 10.1186/s12870-022-03564-2 (PMC8988358; doi:10.1186/s12870-022-03564-2)
Supplement: Supplementary file 1 — Additional file 1. [file Figure S1. The expression profiles of AhSAURs under different stresses..pdf › Figure S1. The expression profiles of AhSAURs under different stresses..pdf]

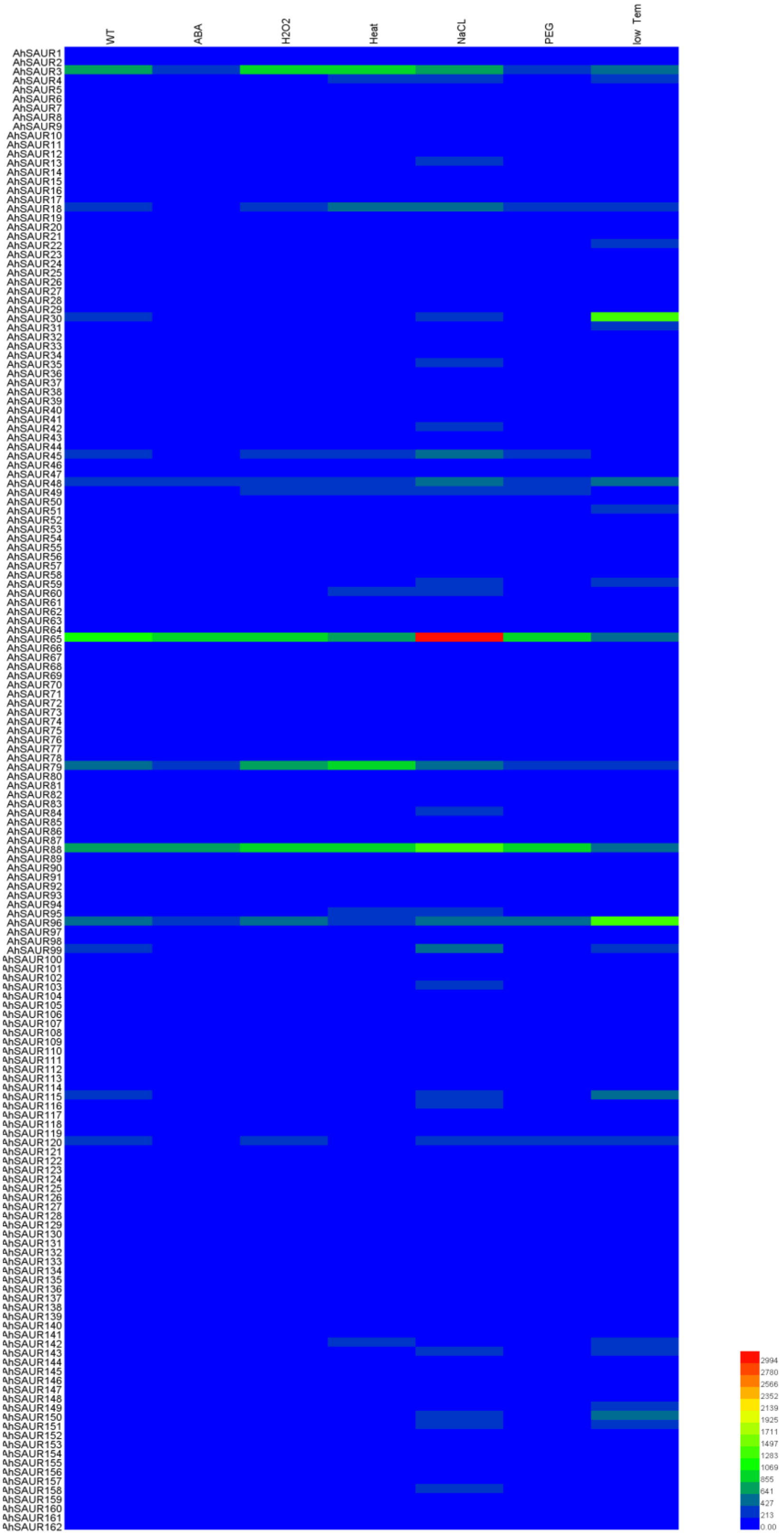

Figure S1. The expression profiles of AhSAURs under different stresses. The gene expression profiles of *AhSAURs* under different stresses., including 75 μM ABA for 24 h, 1% H_2O_2 for 24 h, heat (37 °C) for 24 h, 2% NaCl for 24 h, 20% polyethylene glycol (PEG) 6000 for 24 h, low temperature (4 °C) for 24 h. The expression abundance of each transcript is represented by the normalized fragments per kilobase per million (FPKM) value and displayed as colored boxes from blue (lower expression) to red (higher expression).
